# Environmental and Genetic Factors on the Development of Onychomycosis

**DOI:** 10.3390/jof1020211

**Published:** 2015-08-31

**Authors:** Cerise Adams, Evangelia Athanasoula, Woojung Lee, Nargiza Mahmudova, Tracey C. Vlahovic

**Affiliations:** Department of Podiatric Medicine, Temple University School of Podiatric Medicine, 148 N. 8th Street, Philadelphia, PA 19107, USA; E-Mails: cerise.adams@temple.edu (C.A.); evangelia.athanasoula@temple.edu (E.A.); woojung87@gmail.com (W.L.); tuc72287@temple.edu (N.M.)

**Keywords:** onychomycosis, tinea pedis, environmental factors, age influence of onychomycosis, cultural habits, genetics, trichophyton rubrum, toenails

## Abstract

Since the early 20th century, onychomycosis originated with the onset of war, the use of occlusive footwear, and the mass migration of people by transportation in the United States. Even though onychomycosis has a high prevalence in the US, other parts of the world including Canada, West Africa, Southeast Asia, Northern Australia, and Europe have been well documented with cases of fungal toenail infection in their environments. *Trichophyton rubrum* (*T. rubrum*) is the major dermatophyte responsible for toenail fungal infection and is typically diagnosed in conjunction with tinea pedis, especially in individuals older than 60 years. Gender roles, age, cultural habits, shoe gear, sports activities, and genetic predisposition all contribute to the different presentation of onychomycosis in these areas where organisms like dermatophytes, candida, and molds were isolated in a variety of cases. Despite the differences in isolated pathogens, treatment outcomes remained consistent. This literature review discusses the influence of tinea pedis, genetics, shoe gear, sports, and age on the development of onychomycosis.

## 1. Introduction

Onychomycosis is a fungal infection of the toenails or fingernails that may involve any component of the nail unit. Although not life-threatening, onychomycosis causes an important public health problem due to its increasing prevalence (12% of the U.S. population) [1]. Despite improved personal hygiene and living environments, onychomycosis continues to spread and persist in our society today. Prevalence is increasing in Western countries and is highest in individuals more than 65 years of age [1]. It is often found in conjunction with tinea pedis and sometimes one infection can increase the risk of the other infection [2].

*Trichophyton rubrum* (*T. rubrum*) is the most prevalent pathogen, and an increased incidence of this dermatophyte was observed in toenail onychomycosis. In the US, it is the major pathogen responsible for approximately 90% of toenail onychomycosis cases as well as tinea pedis cases [1]. There are studies that report an autosomal dominant pattern of inheritance associated with *T. rubrum* infection and the increased risk of onychomycosis in subjects where at least one parent had onychomycosis [2].

People with onychomycosis can have serious physical and occupational limitations, which can reduce quality of life due to embarrassment, pain, unsightly appearance, and a negative psychosocial connotation. In 2014, Tosti *et al.* [3] conducted a prospective study of 1655 patients, aged 18 to 70 years, with onychomycosis and found that patients with onychomycosis for >10 years have more psychosocially than physically impaired health-related quality of life (QoL). Of these patients, the greatest psychosocial ramification was directly related to the number of toenails involved.

## 2. Experimental Section

The authors conducted an extensive PubMed literature search, with the following criteria: “(genetics and onychomycosis) and (toenail onychomycosis)”, “(toenail onychomycosis) and (genetics and onychomycosis)”, “(toenail onychomycosis) and (tinea pedis)”, “(tinea pedis) and (toenail onychomycosis)”, “(toenail onychomycosis) and (age)” and “(age) and (toenail onychomycosis)”, “(shoe gear) and (toenail onychomycosis)” and “(toenail onychomycosis) and (shoe gear)”, “(toenail onychomycosis) and (sports)” and “(sports) and (toenail onychomycosis)”. Articles written in a language other than English or prior to the past 10 years were excluded. Original articles and reviews as well as commentaries or correspondence were included. Articles were included if they reported the influence of age, genetics, sports, shoe gear, and environment, as well as tinea pedis, to the development of onychomycosis.

## 3. Results and Discussion

### 3.1. Tinea Pedis and Onychomycosis

The prevalence of onychomycosis of the toenails is highly associated with superficial fungal infections of the skin such as tinea pedis. Szepietowski *et al.* [4] conducted a prospective study in 2006, involving 2761 patients with onychomycosis, and found that 42.8% of the patients had a concomitant fungal infection, with tinea pedis being most common (seen in 33.8% of patients). Interdigital tinea pedis was the most common subtype noted in over 65.4% of cases, followed by heperkeratotic (11.3%) and dyshidrotic (11%) subtypes [4]. It has been hypothesized that in the relationship between onychomycosis and tinea pedis, onychomycosis is the initial infection and tinea pedis serves as the secondary infection as the fungus from the nail unit spreads to the skin [4]. The proximity of the toenails to the rest of the foot appears to be the major reason that there is a larger prevalence between toenail onychomycosis and tinea pedis as compared to other fungal skin infections.

Ali-Shtayeh *et al.* [5] conducted a study of 220 dermatophytosis patients and found that 21.6% of the patients had a concomitant lesions caused by the same dermatophytes at sites distant from the primary lesions in the foot. 13 About 63.2% of patients with tinea pedis had concomitant toenail onychomycosis infection. 13 Therefore, the effective therapy for tinea pedis is to treat the primary infection as well as to prevent spreading of infection to other sites of the skin.

Manual laborers are among the most common groups of people that experience concomitant infections involving tinea pedis and toenail onychomycosis. In 2014, Pichardo-Geisinger *et al.* found that among the Latino manual laborers they surveyed, 32% had onychomycosis and another 37.8% had tinea pedis [6]. The prevalence of a co-infection of onychomycosis and tinea pedis was also determined to be 23.5% [6]. Of the patients who had tinea pedis, 62.05% had onychomycosis [6]. These findings support the hypothesis that tinea pedis along with onychomycosis, both play a major role as sources of infection in the foot.

The concomitant nature of tinea pedis and toenail onychomycosis makes the need to screen for tinea pedis crucial in the presence of toenail onychomycosis and *vice versa*. Lipner *et al.* [7] reviewed a recent *post hoc* analysis of two large studies which showed that treatment of mild to moderate onychomycosis with efinaconazole topical solution 10% was much more effective when co-existing tinea pedis was treated. When tinea pedis was also treated along with onychomycosis, complete cure rates with efinaconazole were 29.4% and mycologic cure rates were 56.2% [7]. When tinea pedis was not treated, complete cure rates were 16.1% and mycologic cure rates were 45.2% [7]. Therefore, it is paramount to initiate treatment when either infection is discovered so as to minimize the risk of developing both. This is especially important in patients that are immunocompromised and less likely to mount an effective host response.

### 3.2. Genetic Polymorphism and Onychomycosis

Onychomycosis is a difficult fungal infection to treat as the opportunity of reoccurrence is high. Many individuals experience chronic onychomycosis as a result of limited cure rates with anti-fungal medications. Dermatophytes may cause infection to the hair, skin and nails of an healthy individual, therefore, environmental exposure is necessary for a fungal infection to develop [8]. However, one must consider the genetic component involved which can make one individual, exposed to the same environmental risk factors, more susceptible to onychomycosis than another. There is increasing evidence that supports the notion that a genetic predisposition may lead to chronic onychomycosis. In 2014, Gupta *et al.* [8] described onychomycosis as an infectious disease; therefore, a genetic component that contributes to the chronicity of the disease is most likely located in the innate or adaptive immune system. The innate immune system is the primary recognition point for fungal infections [8]. Dectin-1, a C-type lectin receptor, identifies the fungal cell wall carbohydrate beta-glucan in dermatophytes and yeasts [8]. It is expressed on macrophages and dendritic cells where it is involved in phagocytosis and the amplification of toll-like receptor (TLR) cytokine production [8]. Dectin-1 increases the signal for tumor necrosis factor alpha (TNF alpha) and independently stimulates the interleukins IL-17, IL-6, and IL-10, which further stimulate the adaptive immune system [8]. A recent study in a family with a high susceptibility to vulvovaginal candidiasis and onychomycosis identified an allele of the Dectin-1 gene containing a single nucleotide polymorphism that results in an early stop codon [8]. The resultant receptor causes deficits in *C. albicans* binding and IL-6 and IL-17 production, which were more pronounced in homozygous individuals but also exist, to a smaller degree, in the heterozygous parental generation [8].

Cell-mediated immunity also plays a crucial role in the development of onychomycosis. There is an association between elevated levels of CD4+CD25+ regulatory T cells (Tregs) and onychomycosis [8]. In a study of 43 onychomycosis patients and 30 healthy controls, the level of CD4+CD25+ Tregs in the peripheral circulation was doubled in those with onychomycosis [8]. A constant increase in Tregs may be associated with the inability of the immune system to clear dermatophyte infections [8]. Additional research is needed to further investigate the role of genetic polymorphism and genotypes in the development of onychomycosis.

### 3.3. The Effects of Shoe Gear and Sports on Onychomycosis

Many authors have proposed a correlation between onychomycosis and the use or disuse of shoe gear. Onychomycosis favors dark, warm, and moist environments. Foot perspiration within a shoe and sock often mimics this setting, making it an ideal atmosphere for fungal maturation. On the other hand, in countries where bare-footed walking through damp areas is the norm among individuals, this too can influence the occurence of onychomycosis [2]. In addition to the above-mentioned, occlusive footwear, a lack of rotation of shoes being worn and exposure to moist environments like health clubs and swimming pools will increase the incidence of onychomycosis [8]. Frequent contact with these sources of infection may initiate a chronic disease state [2].

Sports can also exacerbate the presence of onychomycosis and tinea pedis. A Brazilian study reported a higher prevalence of onychomycosis (two-fold) and concomitant onychomycosis-tinea pedis infections (2.5-fold) in athletes compared to non-athletes [2]. Key predisposing factors contributing to infection in athletes are the speed/intensity involved in running, the sudden starting and stopping nature of the sport (*i.e.*, tennis, soccer, and cricket), practicing sports without protective footwear (*i.e.*, gymnasts, ballet dancers), the frequency of nail injuries, the frequent use of synthetic clothing and shoes that retain sweat, water sports, and communal bathing [2].

### 3.4. Influence of Age on Onychomycosis

Onychomycosis does not affect all age groups equally. The prevalence of onychomycosis was reported in literature to be more common in the elderly. According to Welsh *et al.* [9], prevalence increased up to 30% in patients older than 60 years of age. In more recent studies from Mayo *et al.* [10], they reported prevalence up to 50% in those over 70 years of age. The correlation between increasing age and onychomycosis may be due to several factors. They include reduced peripheral circulation, inactivity, suboptimal immune status, diabetes, larger and distorted nail surfaces, slower growing nails, difficulty in grooming and maintaining foot hygiene, frequent nail injury, and increased exposure to disease-causing fungi in the elderly [2]. A cross-sectional study done by Darjani and colleagues postulated that nursing home care stays attributed to an increase in the risk of developing onychomycosis [11]. Poor vision and arthralgia and, in some cases, physical disability can also make it difficult for the elderly to recognize the disease state leading the fungal infection to progress. In such cases, there is an increased risk of transmission of the pathogens to adjacent nails.

It is generally accepted that onychomycosis is rare in children, although cases in infants can indeed occur. A worldwide prevalence of onychomycosis in children below the age of 18 years is lower than 0.5%, with higher numbers in some countries [2]. In one series of studies done with North American children aged less than 18 years, the prevalence of onychomycosis was 0.16% [12]. A case series study from Peru reports a prevalence of onychomycosis in adolescents aged 12–17 years of 3.4%, which is similar to that found years ago in other tropical countries such as Mexico and Guatemala. The higher prevalence of onychomycosis in children from some countries could possibly be related to the humid environment that facilitates tinea pedis in the feet. In a study data presented from Iceland during the period of 1982–2000, a total of 493 samples from 408 Icelandic children aged 0–17 years was examined. The incidence of positive dermatophyte cultures increased with age and was found in eight children aged 0–4, and in 57 children aged 10–14 years [13].

More research needs to be conducted to determine the possible ways to reinstate the age factors contributing to onychomycosis. A therapeutic intervention that improves the treatment of *T. rubrum* infections would significantly improve the quality of life in patients with chronic mycotic nails. One enhancement in the treatment could be increasing blood flow to the nails of the elderly as well as focusing on earlier treatment of *T. rubrum* infection in younger patients for prevention of long exposure time.

## 4. Conclusions

Understanding the incidence and prevalence of onychomycosis with increasing age, concomitant tinea infection, genetic mutations, sports, and shoe gear is essential for the effective treatment of onychomycosis by physicians. Therefore, it is important not only to treat the lesional toenails but also to address the influence of tinea pedis and environmental factorsFor prevention of future reinfection, it is fundamental to localize all the lesions, identify the causative fungus, establish the prognosis, and choose the most appropriate antifungal agent.
